# Study of the bioremediatory capacity of wild yeasts

**DOI:** 10.1038/s41598-020-68154-4

**Published:** 2020-07-09

**Authors:** Beatriz García-Béjar, María Arévalo-Villena, Eduardo Guisantes-Batan, Juana Rodríguez-Flores, Ana Briones

**Affiliations:** 1 Department of Analytical Chemistry and Food Technology, University of Castilla-La Mancha, Camilo José Cela Avenue, 13071 Ciudad Real, Spain; 2 Regional Institute of Applied Scientific Investigation (IRICA), University of Castilla-La Mancha, 13071 Ciudad Real, Spain

**Keywords:** Fungal biology, Bioremediation

## Abstract

Microbial detoxification has been proposed as a new alternative for removing toxins and pollutants. In this study, the biodetoxification activities of yeasts against aflatoxin B_1_ and zinc were evaluated by HPLC and voltammetric techniques. The strains with the best activity were also subjected to complementary assays, namely biocontrol capability and heavy-metal resistance. The results indicate that the detoxification capability is toxin- and strain-dependent and is not directly related to cell growth. Therefore, we can assume that there are some other mechanisms involved in the process, which must be studied in the future. Only 33 of the 213 strains studied were capable of removing over 50% of aflatoxin B_1_, *Rhodotrorula mucilaginosa* being the best-performing species detected. As for zinc, there were 39 strains that eliminated over 50% of the heavy metal, with *Diutina rugosa* showing the best results. Complementary experiments were carried out on the strains with the best detoxification activity. Biocontrol tests against mycotoxigenic moulds showed that almost 50% of strains had an inhibitory effect on growth. Additionally, 53% of the strains grew in the presence of 100 mg/L of zinc. It has been proven that yeasts can be useful tools for biodetoxification, although further experiments must be carried out in order to ascertain the mechanisms involved.

## Introduction

In recent years, biodetoxification has become a new alternative for the removal of compounds such as microbial toxins, chemical pollutants, and industrial waste products. Depending on the type of system involved, biodetoxification pathways are classified into three categories: (1) commodity-dependent, (2) enzymatic, or (3) microbial^1^.

Microbial detoxification methods may prove useful as tools for providing new ways of eliminating heavy metals or biotoxins, contaminants that have become a growing global concern. Due to industrial development, wastewaters are increasingly being discharged into the environment, either directly or indirectly. Unlike other contaminants, heavy metals are not degradable by natural biochemical pathways. These metals, which tend to accumulate in living organisms, are toxic or carcinogenic to those organisms^2^.

Zinc (Zn) plays a significant regulatory role in several biological processes such as metabolism, where it acts as a cofactor of numerous enzymes and participates in various oxidation–reduction reactions^3^. However, like all heavy metals, Zn may cause negative ecological effects when toxic limits are exceeded, with LD_50_ values in the 186–623 mg Zn/kg/day range, depending on the anion of the salt^4^. Aquatic environments are generally contaminated with large amounts of Zn owing to industrial waste discharge, which can also accumulate in soil sediments. This produces degradation of the ecosystem and biodiversity loss^5^. Different concentrations of Zn can be found in the soil and waters of inhabited areas. Although attempts have been made to clean them using conventional methods, these have proved ineffective when concentrations are below 100 mg/L^6^. Therefore, microbial remediation may constitute a good alternative for mitigating these pollutants. Identifying the strains that tolerate this heavy metal would be of great interest for treating polluted areas^7^ and reducing contamination levels^7,8^.

Mycotoxins are toxic compounds produced by moulds—specifically those of the genera *Aspergillus*, *Penicillium,* and *Fusarium*—that can remain in food products after processing. Exposure to mycotoxins can either occur directly, by eating contaminated food, or indirectly, through animals that have consumed contaminated feed (www.who.int/news-room/fact-sheets/detail/mycotoxins). Aflatoxins, the most dangerous mycotoxins, are classified by the International Agency for Research on Cancer (IARC) as human carcinogens (Category 1). From this group, aflatoxin B_1_ (AFB_1_) is considered to be the most toxigenic and mutagenic example^9^, with oral LD_50_ values ranging from 0.03 to 18 mg/kg/day for most animal species^10^. As estimated by the Food and Agriculture Organization (FAO), 25% of the world’s crop could be affected by mycotoxins, which can unfortunately be found both in human food and animal feed^11^. Aflatoxin contamination is a persistent problem worldwide, and is especially problematic in tropical and subtropical areas, although the Mediterranean area has become prone to aflatoxin contamination due to a shift in traditional occurrence areas caused by climate change^12^. Mycotoxins can be controlled not only directly on the substrate but also through biological control against mycotoxigenic moulds. In the latter case, biological material, such as microorganisms, is used to inhibit the growth of organisms^13^.

Numerous strategies have been developed for solving the environmental and security problems posed by the toxic components outlined above. The main advantage of using yeasts in biodetoxification techniques is that most of the species are safe for living organisms. These yeasts grow on a wide range of substrates and have an ample metabolic diversity.

Studies carried out on the biodetoxification capability of yeast have revealed that some *Saccharomyces* and non-*Saccharomyces* strains are easily able to eliminate heavy metals found in wastewater from the food industry^14-16^. It is also known that yeast cell walls are capable of adsorbing certain mycotoxins during fermentation processes and in final food products^14,17-19^.

Given the background provided above, the purpose of this article is to establish a protocol that would allow an assessment of the detoxification capability of yeast strains isolated from natural ecosystems and to use this approach for selecting the most promising yeasts. Zn, which is especially present in urban wastewaters and has an impact on both aquatic environments and human health, and AFB_1_, a dangerous toxin often leading to food loss and food safety problems worldwide, were selected for this study and their elimination by this technique will be examined.

## Materials and methods

### Yeast strains

A total of 213 yeast strains isolated from different elements, such as flowers, animals, water, and soil, and also from the food industry environment, were studied [unpublished data]. All the isolates were identified at species and strain level in a previous study (Table 1), with a total of 20 different species. The majority of the isolates were from the genera *Diutina*, *Saccharomyces*, *Candida,* and *Rhodotorula*. All yeasts were grown in YPD broth (yeast extract 10 g/L; glucose 20 g/L; peptone 20 g/L), incubated at 30 °C for 24 h, and gently stirred to obtain young cultures.

**Table 1:** Yeast strains studied.

Specie	No. of strains
*Aureobasidium pullulans*	5
*Candida albicans*	27
*Candida intermedia*	3
*Candida magnoliae*	4
*Candida parapsilosis*	8
*Candida sorbophila*	7
*Candida tropicalis*	5
*Candida zeylanoides*	6
*Crytococcus laurentii*	2
*Cystobasidium slooffiae*	1
*Debaryomyces hansenii*	4
*Diutina rugosa*	60
*Exophiala dermatitidis*	1
*Komagataella pastoris*	3
*Meyerozyma guilliermondii*	1
*Minimelanolocus obscurus*	1
*Pichia fermentans*	8
*Pichia kudriavzevii*	9
*Rhodotorula mucilaginosa*	26
*Saccharomyces cerevisiae*	32

### Chemicals and media

AFB_1_ (≥ 98.0% purity) and zinc nitrate (99.0% purity) were purchased from Sigma-Aldrich (U.S.) and Merck (Germany), respectively.

A minimal salts medium (MSM) containing K_2_HPO_4_ 0.4 g, KH_2_PO_4_ 0.2 g, NaCl 0.1 g, MgSO_4_.7H_2_O 0.5 g, MnCl_2_ 0.01 g, Fe(SO_4_)_3_ 0.01 g, and Na_2_MoO_4_ 0.01 g per litre was used for the detoxification assay and the pH was adjusted to 7 as proposed by Abigail and Das^20^. The sterilized medium was supplied with AFB_1_ from a 400 mg/L stock solution in methanol (99.9% purity) or with Zn (Zn(NO_3_)_2_) from a 100 mg/L stock solution in demineralized water. All solutions were pasteurised (75 °C/5 min) or filtered through a cellulose acetate membrane (0.22 µm/diameter) before being added to the MSM. The final concentrations were 0.04 mg/L for AFB_1_ MSM and 1 mg/L for Zn MSM.

### Setting up the detoxification method

*Saccharomyces cerevisiae* (EB62 and EB83) and *Pichia krudiavzevii* (AK8) were the three representative strains chosen in order to identify the best conditions for a reliable and reproducible detoxification protocol. The following parameters were studied:

#### Temperature and contact time

The three strains were inoculated in 25 mL of the defined medium (MSM + toxin) at different temperatures (25 and 30 °C) and for different times (3 and 5 days). The temperature conditions were assayed by establishing a standard time of 5 days for each experiment. The results obtained in this step allowed the temperature to be adjusted and an assessment of the effect of time on cell viability to be made. In total, 12 yeast counts, including duplicates, were carried out for each MSM plus toxin assay.

### Standardisation of the method used for toxin analysis

#### Zinc

Voltammetric measurements were performed with a Metrohm Computrace voltammetric analyser potentiostat (model 757 VA, Eco-Chemie, Utrecht, The Netherlands). A conventional three-electrode system—consisting of an Ag/AgCl/KCl reference electrode, a hanging mercury drop electrode (HMDE) as the working electrode, and a platinum rod as the auxiliary electrode—was used. All measurements were automated and controlled through the programming capacity of the apparatus. The data were treated with a Computrace 757 VA electrochemical analyser.

In order to ascertain whether the yeasts interacted with the electrode, thus causing a lack of sensitivity in the measurements, a comparison between the medium with and without cells was carried out in duplicate. One *S. cerevisiae* strain was grown in MSM + Zn, and the supernatant was obtained by centrifugation (4,500 rpm, 4 min, 10 °C) or filtration through 0.2 µm cellulose acetate membrane (VWR Int., U.S.) for quantification of Zn. These samples were analysed along with those with cells and the results were compared.

To determine whether pH influenced the results, both the direct supernatant (pH 7) and a sample adjusted to normal Zn analysis conditions (pH 2) were measured, with a variation in the ionic forces ranging from 10 to 50 mM. Moreover, different accumulation times (0, 15, and 30 s) and accumulation potentials (from − 1.1 to − 1.5 V) were checked for MSM + Zn, with and without grown strains, to observe where the most sensitive voltammetric signal was obtained. For the adjustment, the tested sample was compared with a standard solution as positive control (1 mg/L Zn solution).

#### Aflatoxin B_1_

For AFB_1_, a 1,260 Infinity HPLC system, coupled to a 6,545 Quadrupole-Time-of-Flight (QToF) spectrometer, was used for the analysis, in conjunction with a mass detector (Agilent, Waldbronn, Germany) and control software (Mass Hunter Workstation; version B.06.11). The analysis parameters and conditions were as described by Iriondo-DeHond et al.^21^. In brief, samples were injected into a Zorbax Eclipse Plus C18 Rapid Resolution HD Column (2.1 × 50 mm, 1.8 μm, Agilent, Santa Clara, CA) with a 5 mm guard column. The temperature was set at 30 °C and the mobile phase was 5 mM ammonium formate + 0.1% formic acid and 5 mM ammonium formate + 0.1% formic acid in methanol. The gradient elution was chosen as indicated in the procedure. Finally, compounds were identified and quantified using the ‘Find by Formula’ algorithm.

Samples were directly centrifuged according to the protocol described by Joannis-Cassan et al.^22^. Different methodologies were proposed for the extraction of AFB_1_: extraction with organic solvents (ethyl acetate or methanol) or the use of a solid phase extraction column (ISOLUTE Myco, Biotage, Sweden). Quantification was achieved by injecting standard solutions from 0.005 to 0.04 mg/L at different sample volumes (10 µL and 30 µL).

### Detoxification assay: determination of residual toxin concentration and cell viability

#### Residual toxin concentration

The best conditions described in the previous section were employed and all yeasts (213) were assayed to ascertain their potential binding capability. Batch experiments were carried out in 100 mL Erlenmeyer flasks containing 25 mL of MSM with each toxin (1 mg/L of Zn and 0.04 mg/L AFB_1_) and then inoculated with 10^6^ cells/mL from overnight cultures. Cell density was measured by microscope count using a Thoma chamber. The samples in the Erlenmeyer flasks were incubated with gentle stirring (150 rpm). Duplicate aliquots were taken at the beginning and at the end of the incubation period for toxin analysis. Two negative controls were also established: MSM without toxin supplied with cell suspension from each strain (NCY) and MSM with each toxin without yeast cells (NCT).

The toxin elimination capability of yeast was calculated using the following formula:

$$ Eliminated\,toxin\,\left( \% \right) = \frac{{ \left( {C_{NCT} - Cs} \right)}}{{C_{NCT} }} \times 100 $$

C_NCT_ refers to ‘toxin concentration from NCT’ and C_S_ to ‘toxin concentration from sample’.

#### Cell viability

To determine whether the presence of toxins affected cellular viability as well as elucidate the possible mechanism of action (adsorption or metabolism), plate counts were carried out on YPD agar (yeast extract 10 g/L, glucose 20 g/L, peptone 20 g/L, agar 20 g/L) using a spiral plater (Eddy Jet 2, IUL Instruments, Barcelona, Spain). Plates were incubated at 30 °C for 2 days and colonies were counted with an automatic counter (Flash & Go, IUL Instruments, Barcelona, Spain). MSM without toxin was inoculated with the tested yeast and the sample was incubated for the same amount of time and at the same temperature to provide a negative control.

### Biocontrol capability against mycotoxigenic moulds

The yeasts with the best detoxification capability were selected and tested on three mycotoxin-forming moulds: *Aspergillus parasiticus* (CECT 2,689), *Fusarium graminearum* (CECT 20,487), and *Penicillium crustosum* (UCLM 93 V). Each mould type was dropped (10^6^ spores/mL) onto the middle of a YPD agar plate. Young yeast cultures (YPD broth at 30 °C during 24 h) were dropped (10^6^ cells/mL) onto the same plate (three yeasts on each plate) and incubated at 30 °C for at least 5 days. Growth inhibition on the mould was observed by comparing the radium of the positive control (mould cultured alone on an agar plate) with the values for the samples with yeasts.

### Tolerance to the presence of Zn in solid media

With the aim of selecting the strains with the best tolerance to Zn in the environment, cell suspensions of young cultures were centrifuged (4,500 rpm/5 min/25 °C) and the pellets were resuspended in YNB broth (Difco-BD, Madrid, Spain) and incubated for 6 h to deplete all sugar reserves. After this time, 10^6^ cells/mL were dropped onto YM agar plates (yeast extract 3 g/L malt extract 3 g/L, peptone 5 g/L, glucose 10 g/L, agar 20 g/L) containing different Zn concentrations (1 mg/L, 25 mg/L, 50 mg/L, 75 mg/L, and 100 mg/L). Plates were incubated at 30 °C for 5 days and the adaptation capability with respect to Zn was observed by evaluating yeast growth.

### Statistical analysis

Statistical analysis was performed using Excel Office 365 software for Windows ver. 2013 (bar graphs) and IBM SPSS for Windows ver. 24 (Student’s t-test, linear regression, analysis of variance [ANOVA] and Duncan test at *p* < 0.005).

## Results and discussion

### Setting up the detoxification method

#### Temperature and contact time

Table 2 shows the best time and temperature conditions. The highest viabilities (log cells/ml) after 5 days were obtained at 30 °C for both MSM. Around 0.5 or 1 log unit was the difference between cells incubated at 25 °C and at 30 °C, as indicated by the Student’s t-test (*p* < 0.005), and significant differences were observed between them in both media. When using 30 °C as the best temperature, counts were 0.5 log units higher at 5 days than at 3 days, with no significant differences (Student’s t-test). Although the only significant differences were observed for temperature conditions, it was detected that incubation time had a less marked effect on the process than temperature. Therefore, the conditions selected were incubation at 30 °C for 5 days because, although counts at 3 days were similar, it appeared that more time was favourable for checking whether toxins were eliminated by secondary metabolism pathways^23,24^ or were bioaccumulated^25^.

**Table 2:** Conditions tested (temperature and time) in both contaminated medias (AFB1 and Zn) and counts (log) obtained (means ± SD).

Strain code	Yeast counts (log)							
	Temperature				Time			
	25 °C		30 °C		3 days		5 days	
	AFB_1_*	Zn*^2^	AFB_1_*	Zn*^2^	AFB_1_	Zn	AFB_1_	Zn
EB62	7.98 ± 0.01	6.47 ± 0.04	8.52 ± 0.01	7.51 ± 0.01	7.97 ± 0.02	7.08 ± 0.03	8.48 ± 0.03	7.57 ± 0.01
EB83	7.91 ± 0.03	6.51 ± 0.15	8.58 ± 0.03	7.13 ± 0.08	7.93 ± 0.03	6.98 ± 0.02	8.55 ± 0.02	7.37 ± 0.07
AK8	6.89 ± 0.03	6.83 ± 0.07	7.21 ± 0.04	7.50 ± 0.01	7.00 ± 0.02	7.33 ± 0.02	7.21 ± 0.02	7.65 ± 0.06

* and *^2^ there are Significant differences between 2 group of samples obtained by Student’s T-test.

### Standardisation of the method used for toxin analysis

#### Zinc

Yeasts incubated in MSM + Zn were treated at three different stages: (1) after a centrifugation step, (2) after a filtration step, or (3) with no treatment. The samples were then analysed by voltammetry with the mercury electrode (Table 3). It was evident that untreated aliquots presented lower sensitivity at the three accumulation times than centrifuged and filtered samples. This behaviour may have been due to the presence of cells interfering with the adequate accumulation and reduction of Zn ions at the mercury electrode. Similar values were obtained in the Zn voltammetry measurements on samples treated by the two methods mentioned above, with slightly higher values obtained for centrifuged samples when compared to filtered samples. Although both techniques showed the same sensitivity, centrifugation was selected as the cell removal treatment in order to standardise the method for both MSM supplied with toxins.

**Table 3:** Determination Zn concentration (means ± SD) at different cell elimination treatments.

Treatment of the samples	Zn (mg/L)		
	T0s	T15s	T30s
Positive control	1.00 ± 0.00	1.00 ± 0.00	1.00 ± 0.00
No treatment (directly)	0.42 ± 0.17	0.50 ± 0.05	0.42 ± 0.08
Filtered	0.70 ± 0.03	0.67 ± 0.02	0.70 ± 0.07
Centrifuged	0.71 ± 0.01	0.70 ± 0.03	0.71 ± 0.06

Regarding the adjustment of ionic strength in the potassium phosphate buffer used for the voltammetry measurements, different ionic buffer strengths were assessed (from 10 to 50 mM) at pH 7 in order to identify the most sensitive signal. An ionic strength of 10 mM in the pH 7 buffer yielded the most accurate and sensitive Zn measurements. Accumulation times were evaluated by testing representative samples at 0, 15, and 30 s (Table 4). It was observed that the Zn concentrations (mg/L) for EB83 were 0.28, 0.31, and 0.31 at the three times used (0 [T0s], 15 [T15s], and 30 [T30s], respectively) and for EB62 the values were 0.63, 0.74, 0.73 at T0s, T15s, and T30s, respectively. These results indicate that an accumulation time of T0s showed a slight loss of sensitivity for Zn detection, while at T15s and T30s the measurements were very similar. The aforementioned loss was more acute for samples that contained higher Zn concentrations. All three tests showed optimal sensitivity, but, in order to save time, T15s accumulation was preferred, as it not only provides quick measurement but also does not present a loss of sensitivity. If necessary, in future studies, a second measurement at a higher accumulation time could be carried out for samples with lower Zn concentrations (e.g., 1 µg/L or 1 ng/L). The accumulation potentials for the Zn measurements were checked from – 1.1 to – 1.5 V and the calibration was based on the peak reduction signal, so a potential with less signal noise was chosen for the analysis. In this case, an accumulation potential of – 1.2 V was selected.

**Table 4:** Determination Zn concentration (means ± SD) at different times of accumulation: 0 (T0s), 15 (T15s) and 30 s (T30s).

Strain code	Zn (mg/L)		
	T0s	T15s	T30s
Positive control	1.00 ± 0.02	1.00 ± 0.01	1.00 ± 0.01
EB62	0.63 ± 0.01	0.74 ± 0.01	0.73 ± 0.03
EB83	0.28 ± 0.09	0.31 ± 0.01	0.31 ± 0.05

Based on the above results, the conditions selected for the quantification of residual Zn after the detoxification assay were as follows: centrifugation was chosen as the cell-removal technique, as it not only speeds up the process but also reduces the time and material consumed; 10 mM was selected as the ionic strength of the buffer; 15 s was selected as the accumulation time; and, finally, – 1.2 V was employed as the accumulation potential.

#### Aflatoxin B1

From the three options tested for extraction, solid phase extraction columns constitute a good extraction method, but this method was impractical for a large number of samples, so it was ruled out for this experiment. Additionally, bibliographic research revealed that, although methanol is used for the extraction of other mycotoxins^17^, in aqueous samples ethyl acetate proved to have a higher extraction efficiency than methanol^26^. It is also a cheap low-toxicity solvent. Therefore, the same volume of supernatant and ethyl acetate (PanReac, Barcelona, Spain) were mixed for 1 min and the ethyl acetate phase was collected. AFB_1_ suspensions were concentrated using a SpeedVac concentrator (Thermo Savant ISS110, New York, U.S.) and resuspended in a 20% Methanol/80% MilliQ water (v/v) solution. The six-point calibration curve allowed the high sensitivity and reproducibility of the method to be confirmed. All points were detected at both sample volume injections (10 µL and 30 µL), but 30 µL was selected as the injection volume based on previous studies on AFB_1_ quantification^21^.

### Detoxification assay: determination of residual toxin concentration and cell viability

#### Residual toxin concentration

**Figure 1:**
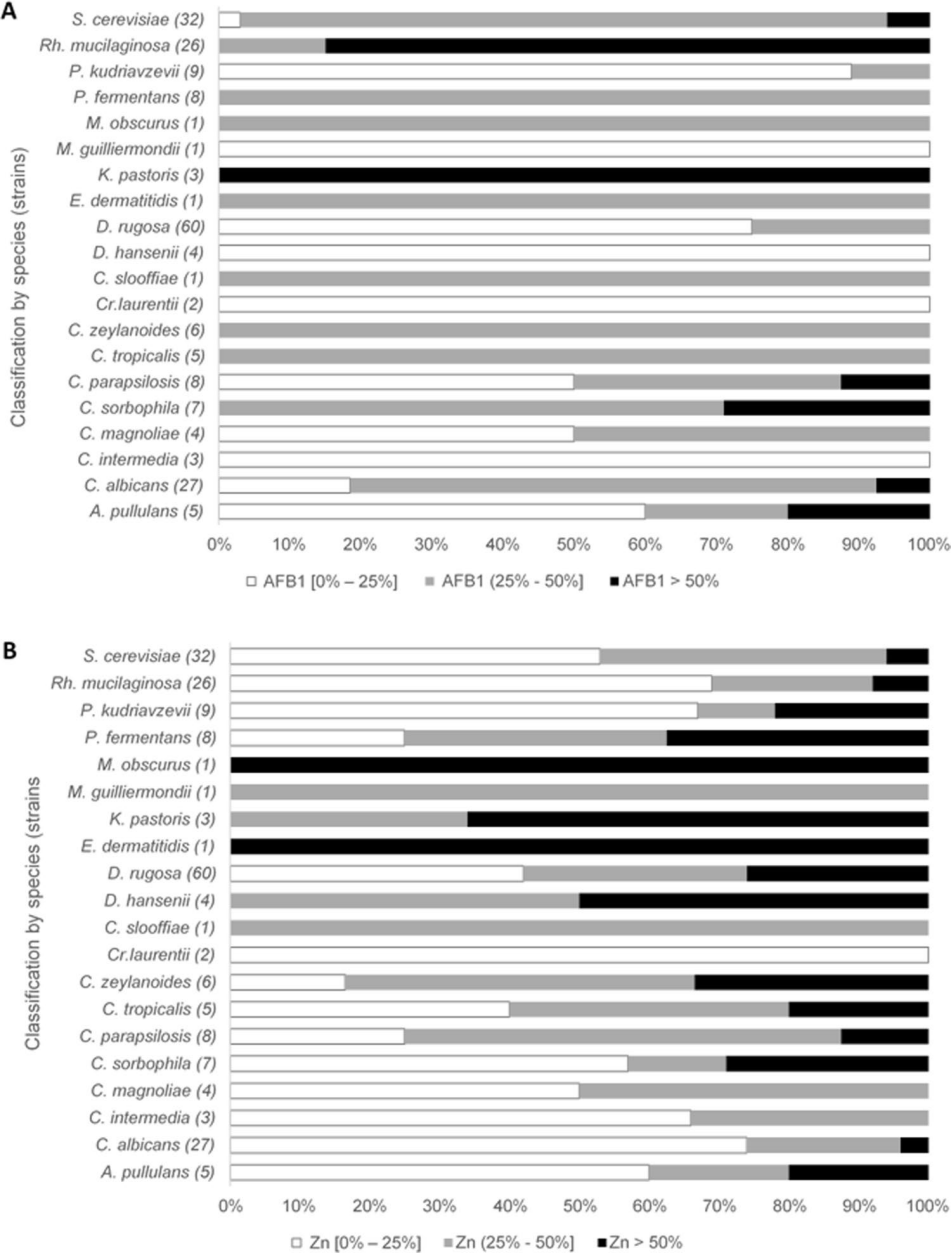
Classification of yeast strains depending on its biodetoxification capability (0–25%; 25–50%; > 50% of elimination) of AFB_1_ (**A**) or Zn (**B**).

The yeast strains (213) were inoculated in both MSM + Zn and MSM + AFB_1_ to evaluate detoxification capability. The yeasts were grouped into three different sets according to toxin elimination percentage: (1) 0–25% elimination, (2) 25–50% elimination, and (3) over 50% elimination (Fig. 1). Detoxification behaviour was observed to be different between AFB_1_ and Zn. Over 50% of the yeasts were able to eliminate AFB_1_. A large number of strains (102) detoxified AFB_1_ by 25% to 50%, and 33 strains were able to remove over 50%. Regarding Zn, although 106 of the tested strains were not capable of eliminating more than 25%, 39 strains eliminated over 50% of the Zn. The 66 strains that eliminated over 50% of the toxins are listed in Table 5. *Rh. mucilaginosa* was the species with the most strains (22) with a high capability for AFB_1_ elimination (around 65%), although it did not present the same efficiency against Zn, as only two strains were able to remove over 70% of the Zn.

**Table 5:** Classification of the strains with detoxification capability > 50% of toxin removed (A: AFB_1_; B: Zn; means ± SD).

A			B		
Yeast species	Strain code	% AFB_1_ removed	Yeast species	Strain code	% Zn removed
*A. pullulans*	H1	68.61 ± 0.67^g,h,i^	*A. pullulans*	H1	91.14 ± 2.39^i,j,k^
*C. albicans*	AA17	50.34 ± 1.74^a^	*C. albicans*	AG3	60.86 ± 14.34^a,b^
	AA19	64.61 ± 0.91^f,g,h,i^	*C. parapsilosis*	ECF6	56.33 ± 0.45^a^
*C. parapsilosis*	ECF42	55.37 ± 3.29^a,b,c,d,e^	*C. sorbophila*	ECF12	71.58 ± 6.78^a,b,c,d,e,f,g,h,i^
*C. sorbophila*	ECF16	52.59 ± 5.45^a,b,c^		ECF85	59.78 ± 0.86^a,b^
	ECF85	51.49 ± 0.06^a,b^	*C. tropicalis*	AK11	68.65 ± 2.80^a,b,c,d,e,f^
*K. pastoris*	EW1	71.50 ± 3.53^i^	*C. zeylanoides*	ECF5	76.605 ± 2.134^b,c,d,e,f,g,h,i,j^
	EW3	51.50 ± 2.12^a,b^		ECF78	69.765 ± 3.952^a,b,c,d,e,f,g^
	EW6	50.50 ± 2.12^a,b^	*D. hansenii*	AB1	72.88 ± 6.53^a,b,c,d,e,f,g,h,i^
*Rh. mucilaginosa*	AA14	52.77 ± 3.29^a,b,c,d^		AB8	84.32 ± 6.68^d,e,f,g,h,i,j,k^
	AB7	67.91 ± 1.03^g,h,i^	*D. rugosa*	EB8	74.45 ± 6.55^a,b,c,d,e,f,g,h,i^
	AG16	67.70 ± 0.24^g,h,i^		EB84	69.41 ± 2.16^a,b,c,d,e,f,g^
	AG17	62.37 ± 0.55^e,f,g,h^		ECF52	88.68 ± 10.67^g,h,i,j,k^
	AS1	57.16 ± 0.33^b,c,d,e,f^		ECF53	64.88 ± 0.86^a,b,c,d^
	AS2	62.81 ± 0.50^e,f,g,h,i^		ECF59	81.44 ± 1.49^c,d,e,f,g,h,i,j,k^
	AS6	62.86 ± 0.68^e,f,g,h,i^		ECF61	60.67 ± 6.12^a,b^
	AS7	63.58 ± 1.90^e,f,g,h,i^		ECF72	70.32 ± 7.74^a,b,c,d,e,f,g^
	AS8	61.46 ± 0.21^d,e,f,g,h^		ECF76	66.32 ± 6.63^a,b,c,d,e^
	AS9	60.97 ± 2.37^c,d,e,f,g^		FC18	79.06 ± 2.13^b,c,d,e,f,g,h,i,j^
	AS12	63.64 ± 1.59^e,f,g,h,i^		FC19	77.92 ± 4.62^b,c,d,e,f,g,h,i,j^
	EB12	60.69 ± 0.70^c,d,e,f,g^		FP5	98.49 ± 0.00^k^
	EB14	66.55 ± 0.32^g,h,i^		FP6	63.96 ± 0.26^a,b,c^
	EB16	70.20 ± 1.18^h,i^		FP11	90.94 ± 5.86^h,i,j,k^
	EB25	68.35 ± 0.09^g,h,i^		FP20	80.82 ± 4.68^c,d,e,f,g,h,i,j,k^
	EB39	67.29 ± 1.03^g,h,i^		FR4	65.34 ± 6.42^a,b,c,d^
	ECF46	66.56 ± 0.08^g,h,i^		FR19	67.35 ± 0.79^a,b,c,d,e^
	ECF50	60.96 ± 3.76^c,d,e,f,g^	*E. dermatitidis*	AB13	68.00 ± 5.65^a,b,c,d,e,f^
	ECF90	65.52 ± 0.64^f,g,h,i^	*K. pastoris*	EW1	71.50 ± 3.47^a,b,c,d,e,f,g,h^
	ECF107	64.98 ± 0.19^f,g,h,i^		EW3	60.18 ± 4.04^a,b^
	EW2	60.68 ± 0.21^c,d,e,f,g^	*M. obscurus*	AG19	85.220 ± 6.037^e,f,g,h,i,j,k^
	EW5	60.49 ± 0.82^c,d,e,f,g^	*P. fermentans*	ECF32	90.82 ± 2.97^h,i,j,k^
*S. cerevisiae*	EB34	52.25 ± 3.57^a,b^		ECF43	72.02 ± 0.65^a,b,c,d,e,f,g,h,i^
	EB57	51.12 ± 4.66^a,b^		ECF55	74.31 ± 1.67^a,b,c,d,e,f,g,h,i^
			*P. kudriavzevii*	AK9	77.17 ± 0.99^b,c,d,e,f,g,h,i,j^
				ECF30	75.88 ± 2.49^a,b,c,d,e,f,g,h,i^
			*Rh. mucilaginosa*	ECF107	69.44 ± 5.23^a,b,c,d,e,f,g^
				EW2	87.65 ± 0.83^f,g,h,i,j,k^
			*S. cerevisiae*	EB21	95.56 ± 0.57^j,k^
				EB83	70.24 ± 2.16^a,b,c,d,e,f,g^

The different letters of the superscript indicate significant differences (p ≤ 0.05 per column).

*Aureobasidium pullulans* (H1) presented good behaviour with both toxins (almost 70% of AFB_1_ and over 90% of Zn). Nevertheless, some species can eliminate over 90% of Zn but not AFB_1_, e.g., *S*. *cerevisiae* (EB21). The opposite behaviour was observed for EB16 (*Rh. mucilaginosa*), which was able to absorb 70% of the AFB_1_ but eliminated less than 20% (data not shown). This would mean that the detoxification ability of strains is different depending on the toxin. Moreover, strain FP5 (*D. rugosa*) showed strong activity against Zn (nearly 100% removal), but it did not show any detoxification activity against the mycotoxin. The three *K. pastoris* (EW1, EW3, and EW6) strains exhibited good capability against the two toxins, with high percentages of elimination. Overall, strains from different species showed different behaviours against the two toxins, so this activity is clearly strain-dependent.

**Figure 2:**
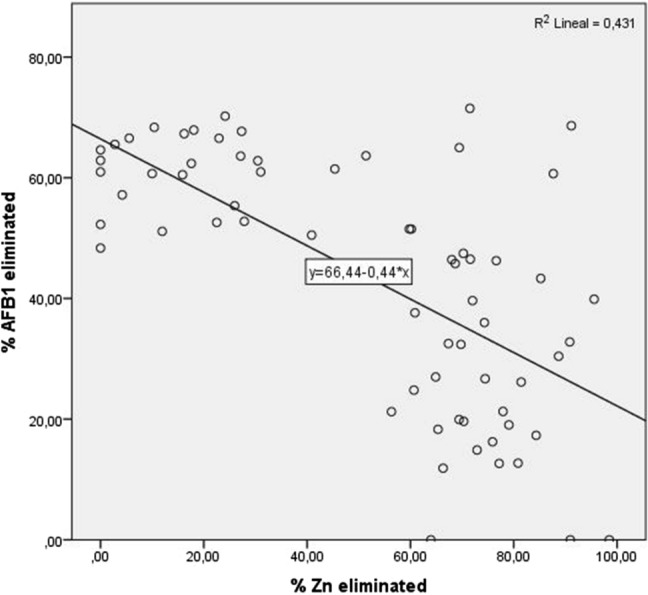
Relationship between the capability of AFB_1_ and Zn elimination in the yeasts with uptake higher than 50%.

The use of yeast for mycotoxin detoxification has been reported previously by other authors^22,27^. It was shown that the percentage of mycotoxins adsorbed by yeast varied depending on the strain, with *Rh. mucilaginosa* strains removing higher percentages of other mycotoxins. In contrast, heavy metal detoxification by yeast has rarely been reported and most studies have been carried out with *S. cerevisiae* species or *Candida sp*. McCormick^28^ showed that removal percentage is associated with the strain. Statistical analysis was focused on the strains that could remove over 50% of AFB_1_ or Zn, showing that capability is also toxin-dependent (Fig. 2). The low linear regression rate (0.431) indicates that strains that can remove certain compounds are not necessarily capable of removing other toxins in the same way. However, the toxins tested in this experiment are chemically different, so this behaviour would vary depending on biochemical structure. Moreover, as can be seen from the results in Table 5, the percentage of AFB_1_ bound by strains did not exceed 71.5%, whereas for Zn it was almost 99%. In addition, of all the strains tested, 39 were able to eliminate over 50% of the Zn present in the media, but fewer strains (33) were able to achieve the same level of decontamination with the AFB_1_-contaminated media. Based on these two findings, ANOVA analysis (*p* < 0.005) indicated that the number of groups with significant differences in the Zn detoxification test is greater than in the AFB_1_ test.

#### Cell viability

All the strains tested were able to grow in contaminated MSM after 5 days at 30 °C. Generally, yeast counts indicated a concentration increase of 0.5–1 log unit at the end of the assay, i.e., showing the same amount of growth as the negative controls. However, certain strains, mostly from *D. rugosa*, were found to have a lower growth rate (0.1–0.2 log units) compared to the negative control (1 log unit; data not shown).

**Figure 3:**
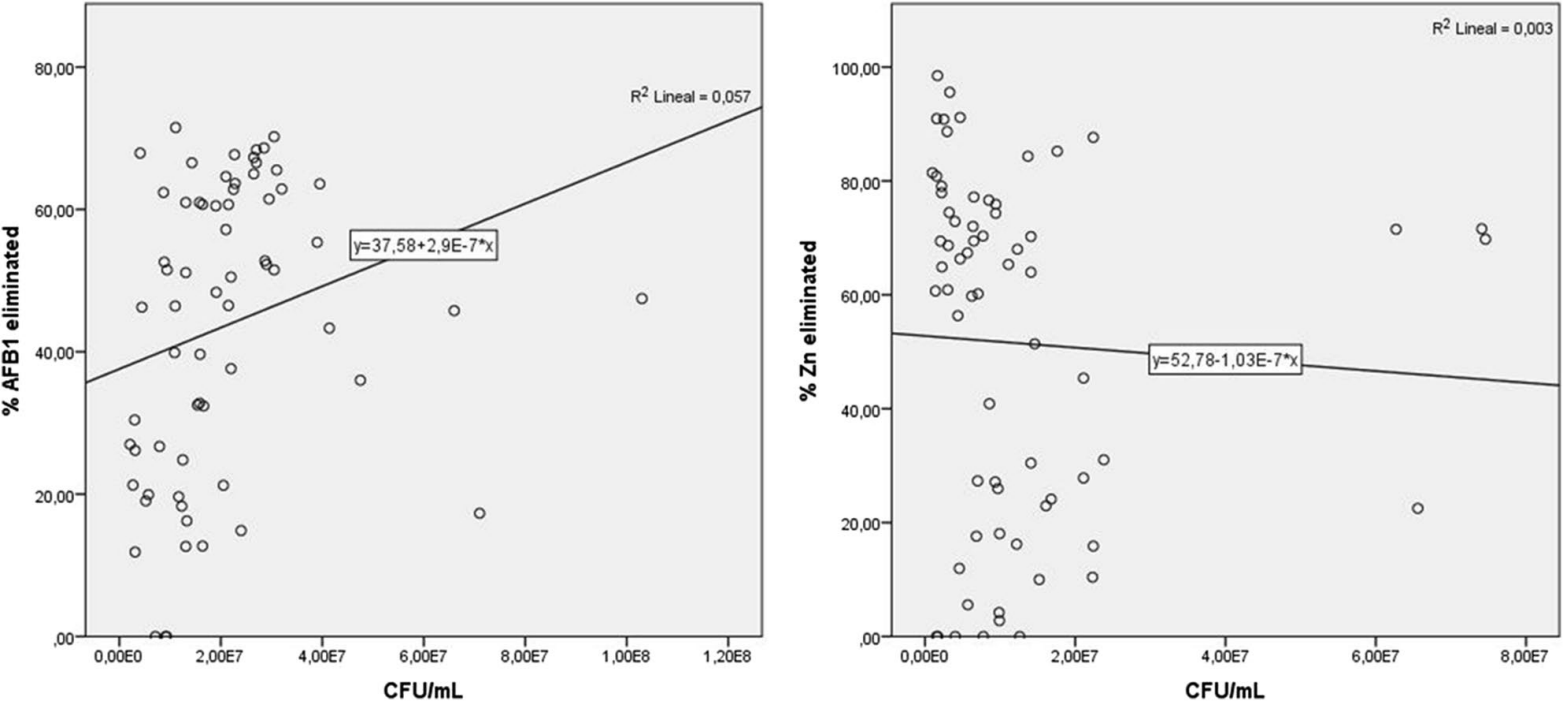
Relationship between the capability of AFB_1_ (**A**) or Zn (**B**) elimination and yeasts count with uptake higher than 50%.

The relationship between biomass growth and toxin elimination, as evaluated by linear regression analysis (0.003; 0.057), showed that toxin binding by yeast is not related to log unit increase (Fig. 3). Therefore, in general, the strains tested were not able to use the contaminated media for their development, which probably indicates that yeasts eliminate toxins by cellular adsorption. Aflatoxins are known to be diminished by physical binding rather than degradation in some microorganisms^29^ and heavy metal bioremediation is normally carried out in yeast by bioaccumulation in the vacuoles^29^ or by biosorption in cell walls^29,30^. In any case, the elimination mechanism of these strains should be studied thoroughly in future projects.

#### Biocontrol capability against mycotoxigenic moulds

The yeast strains that eliminated over 50% of the toxins, either AFB_1_ or Zn, were grown on the same plate as a mycotoxigenic mould (*A. parasiticus*, *F. graminearum,* and *P. crustrosum*).

**Figure 4:**
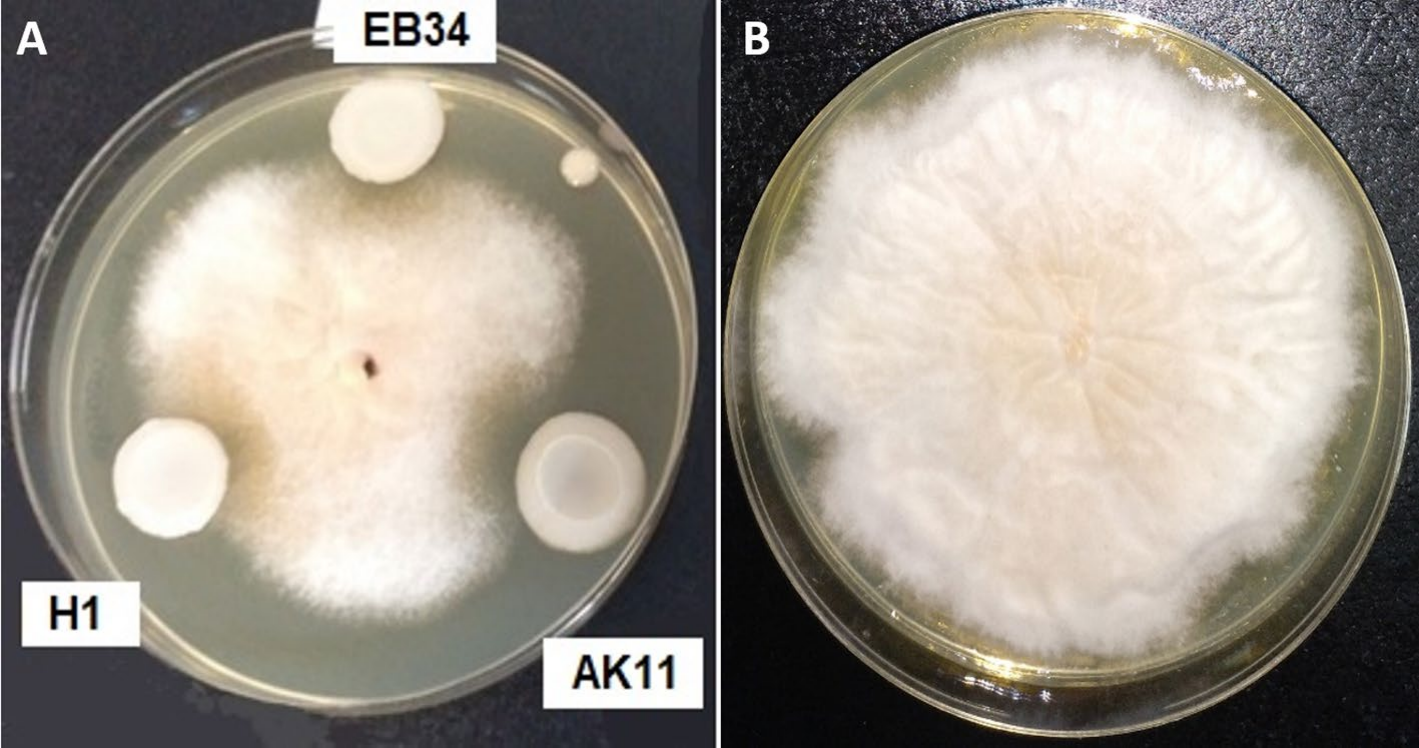
Biocontrol activity of *Saccharomyces cerevisiae* (EB34), *Candia tropicalis* (AK11) and *Aerobasium pulullans* (H1) against *F. graminearum* after 5 days of incubation at 30 °C (**A**) compared with the mould control (**B**).

Of all the strains (66) tested in this assay, 48.5% had varying intensities of biocontrol activity and were effective against at least one of the moulds (Table 6). Twenty-three of the strains affected the growth of *A. parasiticus* and these were AK11 (*C. tropicalis*), EW1 (*K. pastoris*), H1 (*A. pullulans*), EB35 (*Rh. mucilaginosa*), and ECF42 (*C. parapsilopsis*) strains. All of these strains reduced the mycelium by 50 to 60% and they showed significant differences from other strains. The activity presented against *F. graminearum* was weaker. Although 18 strains showed activity, only three presented over 50% inhibition, namely AK11 (*C. tropicalis*), EW3 (*K. pastoris*), and AS6 (*Rh. mucilaginosa*)*.* Of these, AK11 showed the best results, with a reduction of 64.3%, and was classified in the group with the largest significant difference with respect to the negative control. An example of the mycelium inhibition of this mould is shown in Fig. 4. Only 11 of the strains were able to inhibit *P. crustrosum*. The best examples (over 50% inhibition) were ECF59, FR19 (*D. rugosa*), EB83 (*S. cerevisiae*), AB7 (*Rh. mucilaginosa),* and ECF42 (*C. parapsilopsis)* (Table 6). Finally, in all cases, yeasts with biocontrol capability showed significant differences with respect to the negative controls. Some of the yeasts (ECF42-*C. parapsilopsis*, EW3-*K. pastoris,* and EB83-*S. cerevisiae*) showed inhibitory activity against the three moulds, albeit with different intensities.

**Table 6:** Biocontrol activity of yeast strains on *A. parasiticus*, *F. graminearum* and *P. crustrosum* (growth radius after 5 incubation days; means ± SD).

Yeast species	Strains code	Mycelium radius (mm)		
		*A. parasiticus*	*F. graminearum*	*P. crustrosum*
	Negative control^1^	31.3 ± 0.8^l^	41.5 ± 0.4^l^	25.9 ± 0.5^g^
*A. pullulans*	H1	14.8 ± 1.8^a,b,c^	26.3 ± 1.1^e,f^	NI^2^
*C. albicans*	AA17	21.0 ± 1.4^f,g,h^	NI	NI
	AA19	17.8 ± 1.8^c,d,e,f^	37.0 ± 1.4^j,k^	NI
	AG3	27.3 ± 1.1^k^	35.3 ± 0.4^i,j^	NI
*C. parapsilosis*	ECF42	15.8 ± 1.8^a,b,c,d^	24.3 ± 0.8^d,e^	13.5 ± 0.8^b,c,d^
*C. sorbophila*	ECF12	25.1 ± 1.8^i,j,k^	NI	15.8 ± 0.4^d^
	ECF16	26.9 ± 0.8^k^	NI	NI
	ECF85	25.4 ± 1.2^j,k^	NI	NI
*C. tropicalis*	AK11	12.8 ± 0.4^a^	14.8 ± 1.1^a^	NI
*D. rugosa*	ECF53	20.4 ± 1.6^e,f,g,h^	NI	NI
	ECF59	17.4 ± 1.2^b,c,d,e^	NI	8.2 ± 1.8^a^
	ECF61	21.8 ± 0.4^g,h,i^	33.8 ± 0.8^h,i^	NI
	FR4	NI	25.4 ± 0.9^d,e^	NI
	FR19	NI	27.9 ± 1.6^f^	11.2 ± 2.5^a,b,c^
	FP6	24.8 ± 1.0^i,j,k^	24.6 ± 0.4^d,e^	NI
	FP11	NI	25.0 ± 1.1^d,e^	NI
*K. pastoris*	EW1	14.1 ± 2.2^a,b^	38.9 ± 0.8^k^	NI
	EW3	19.8 ± 0.4^e,f,g,h^	23.5 ± 0.7^c,d^	21.2 ± 2.2^e,f^
	EW6	21.9 ± 0.8^g,h,i,j^	18.3 ± 1.1^b^	NI
*Rh. mucilaginosa*	AB7	NI	NI	10.30 ± 0.00^a,b^
	AG17	NI	25.7 ± 0.5^d,e^	NI
	AS1	NI	NI	14.5 ± 2.3^c,d^
	AS2	NI	23.7 ± 0.8^c,d^	NI
	AS6	NI	21.9 ± 0.8^c^	24.4 ± 2.1^f^
	AS9	NI	NI	19.9 ± 1.8^e^
	EB25	15.1 ± 1.6^a,b,c,d^	NI	20.25 ± 0.49^e^
	ECF46	22.9 ± 1.9^h,i,j^	NI	NI
	ECF49	22.5 ± 0.8^h,i,^	NI	NI
*S. cerevisiae*	EB21	18.0 ± 0.7^c,d,e,f^	NI	NI
	EB34	18.5 ± 0.7^d,e,f,g^	NI	NI
	EB57	17.5 ± 2.1^b,c,d,e,f^	30.8 ± 1.1^g^	NI
	EB83	17.3 ± 0.3^b,c,d,e^	32.3 ± 1.1^g,h^	10.8 ± 0.4^a,b^

The different letters of the superscript indicate significant differences (p ≤ 0.05) per column.

^1^Negative control: mycotoxigenic fungi cultured alone on the plate.

^2^NI, no inhibition.

Biocontrol activity was assessed as another strain-dependent capability. Strains from the same species exhibited different behaviours against the three moulds, in varying intensities. *C. albicans* strains showed the same behaviour against these moulds, but strains from other species, such as *K. pastoris*, *D. rugosa*, and *Rh. mucilaginosa*, presented different levels of action against the mycotoxigenic moulds (Table 6). Studies have indicated that biocontrol activity in some microorganisms against others can be caused by competition for media nutrients or due to the antifungal secretions they produce in the media^28^. Zymocines produced by some species, such as *S. cerevisiae* and *D. hansenii,* are a common mechanism of action. These toxins provide a new tool that may be an alternative for synthetic fungicides^31−33^. The production of extracellular enzymes (β-glucanases and chitinases) by *A. pullulans* has proven to be an effective pathway against moulds^34^ and this could be the reason why strain H1 showed this ability. *A. pullulans* has previously been used as a biocontrol agent for postharvest crop diseases caused by *Botrytis cinerea*^35^. Furthermore, Sperandio et al.^36^ found that an *A. pullulans* strain isolated from plants was capable of reducing the mycelium growth by 30–41.2%, a range of action similar to that observed in this article, although the mechanism of action could not be determined. Likewise, it has been reported that *Rh. mucilaginosa* strains isolated from peach blossoms reduce blue and grey mould decay on treated fruits, with an almost complete inhibition (97.2 and 97.1%) achieved when higher yeast cell concentrations (10^9^ cells/mL) are used^37^. However, in this study, the *Rh. mucilaginosa* strain that showed the best biocontrol activity was adjusted to 10^6 ^cells/mL, and better results may be expected if a higher concentration is used. Other pieces of research have identified some species of *Candida* and *Pichia sp,* which, isolated from natural sources, presented biocontrol activity in vivo in fruit^33,34,38,39^.

In the study reported here, the AK11 (*C. tropicalis*) strain proved to have the best biocontrol activity against both *A. parasiticus* (57.4%) and *F. graminearum* (64.3%), although it did not have any effect on *P. crustrosum* growth. ECF59 (*D. rugosa*) showed the best mycelium reduction of *P. crustrosum* (68.3%) and it also reduced the growth of *A. parasiticus* by 44%, although it did not affect the *Fusarium* fungi.

#### Tolerance to the presence of Zn in solid media

**Figure 5:**
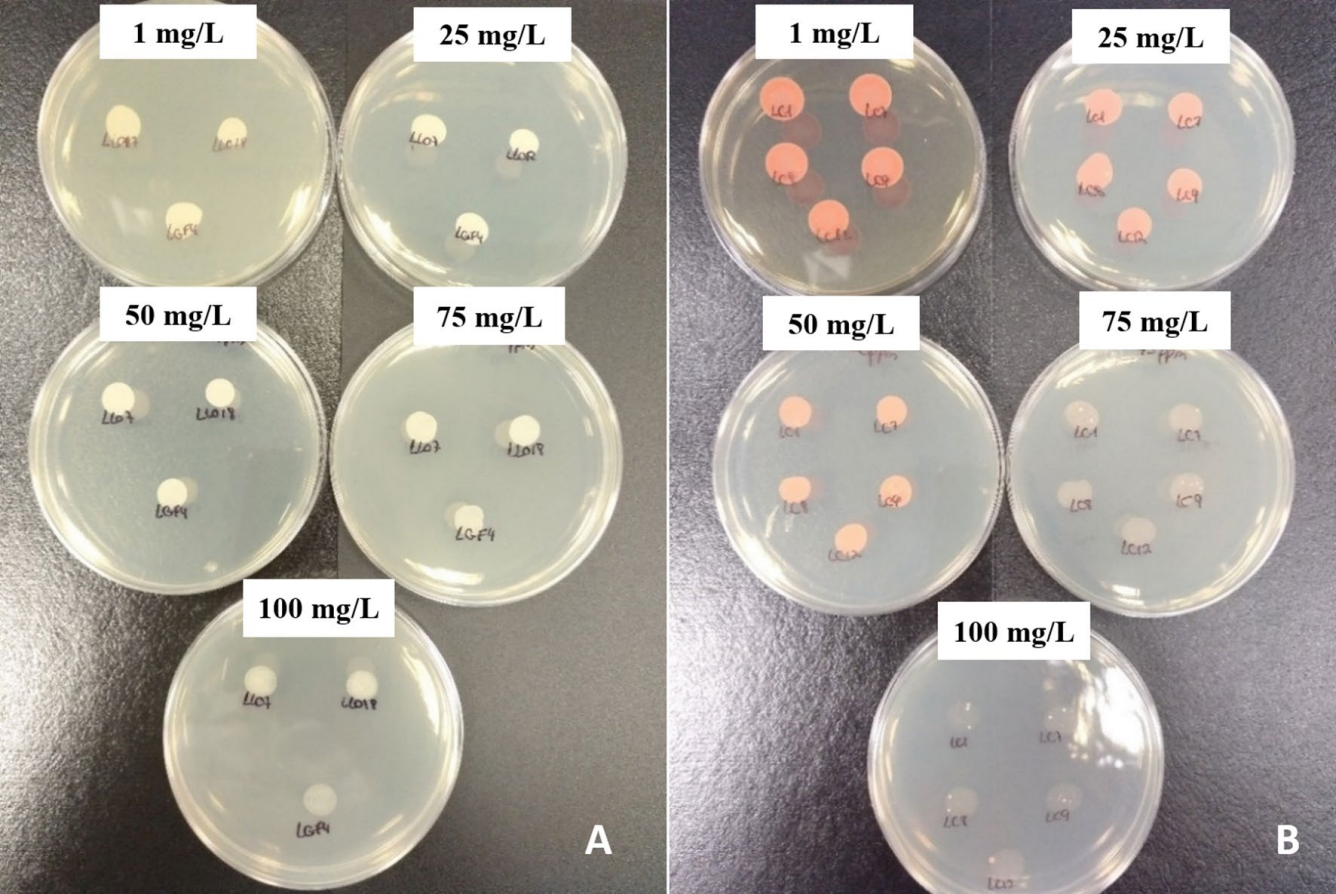
*Pichia fermentans* strains (**A**) and *Rh. mucilaginosa* strains (**B**) growth on YM agar supplemented with different concentrations of Zn (1, 25, 50, 75 and 100 ppm).

In order to evaluate tolerance to Zn, the 66 strains that showed the best biodetoxification capability against both toxins were cultured in YM agar supplemented with different concentrations of Zn. After the incubation time, the samples were visually assessed and their growth identified as weak or strong. The results are provided in Fig. 5. Microbial growth occurred with up to 50 mg/L of Zn salt in 75% of the cases, and 53% of the yeasts tolerated the highest concentration (100 mg/L). As expected, all strains resisted the lower concentrations as they did in the detoxification assay.

**Figure 6:**
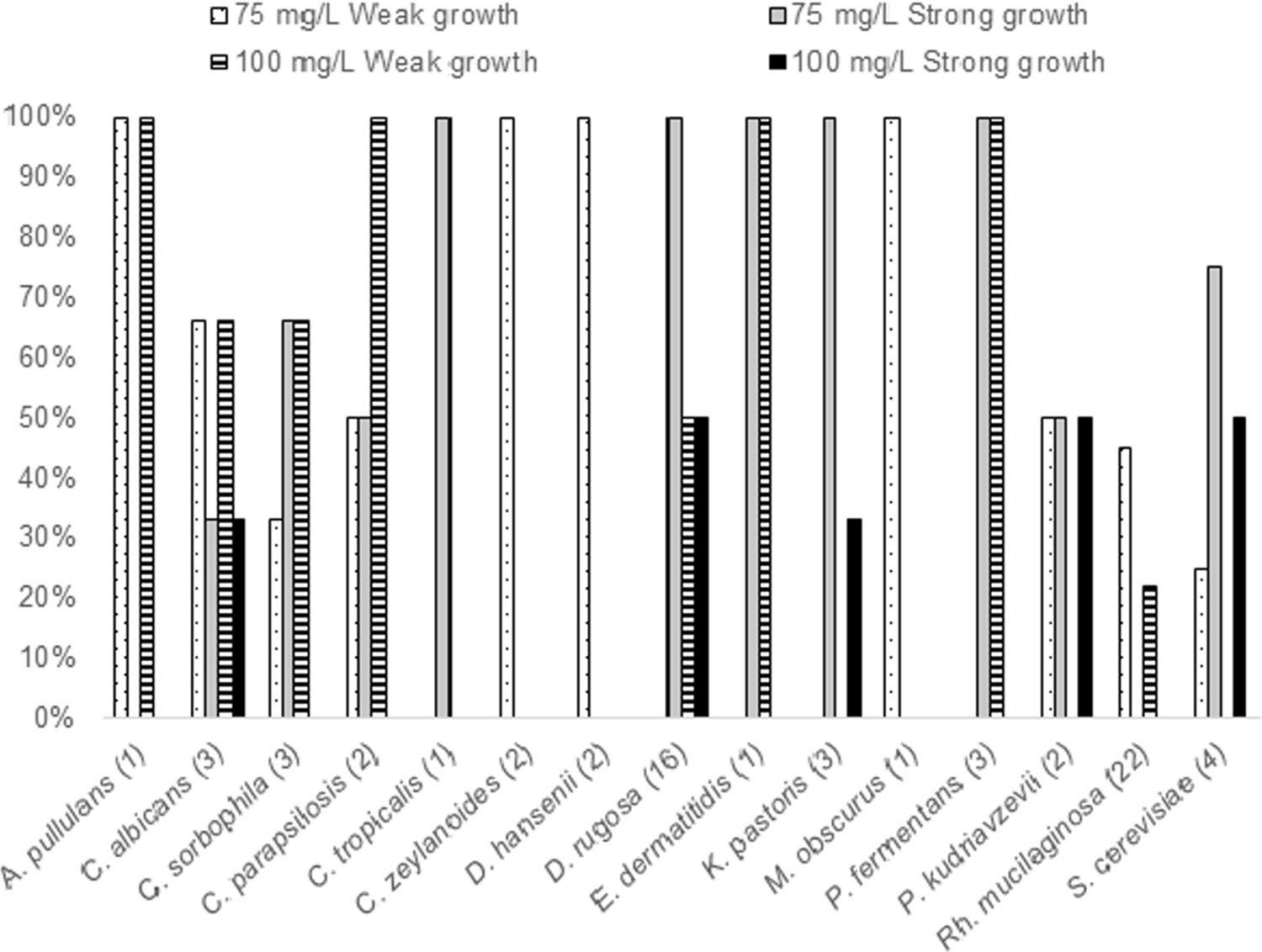
Distribution (%) of strain studied with capability for growing at 75 and 100 ppm of Zn.

The results of these experiments are shown in Fig. 6. Strains with weak growth at 75 mg/L showed the same behaviour or zero growth at 100 mg/L, e.g., *Rh. mucilaginosa*. In contrast, strains with good growth at 75 mg/L were capable of resisting concentrations of 100 mg/L and showed a different development. The yeasts with the best behaviour (strong growth at 100 mg/L) were *C. albicans*, *D. rugosa*, *K pastoris*, *P. kudriavzevii*, and *S. cerevisiae*.

Muñoz et al.^40^ published similar results for *Rh. mucilaginosa*, which could be the reason why its strains presented low biodetoxification activity against this heavy metal. Furthermore, some strains of *D. rugosa* such as FP5, which showed strong growth at 100 mg/L Zn, was able to almost completely eliminate (98.4%) the Zn present in the minimum salt medium, thus showing that tolerance to the ion is a key factor for biosorption. The only *A. pullulans* strain (H1) tested tolerated the highest Zn concentration, as documented previously^41^. Yeasts from the *Pichia* and *Candida* genus were also described as Zn-resistant and were able to tolerate up to 1.3 mg/L (20 mM)^38^. Similarly, various studies have indicated different levels of Zn resistance in *S. cerevisiae*. While some strains showed sensitivity, others were reported to resist concentrations of up to 0.065 mg/L (1 mM) of Zn^40−43^, thus supporting the heterogeneous results obtained with *S. cerevisiae* strains in the study reported here. In addition, Castro-Silva et al.^44^ reported that all tested yeast strains isolated from a coal mine were able to resist different concentrations of Zn. Although microorganisms isolated from contaminated environments tend to be more tolerant to these pollutants, other studies have indicated that there is very little difference in metal tolerance between strains from polluted and unpolluted sites^45^. Thus, the strains used in this experiment proved to have a good Zn tolerance, similar to those reported in other studies. Resistance to Zn by yeasts with good bioremediation capacity is important since this metal can be found in a wide range of concentrations in contaminated environments. Once again, the heterogeneity of the results showed that the response of the isolates to heavy metals essentially depended on the strain and the heavy metal concentration, as previously indicated by Muñoz et al.^40^.

In conclusion, the results reported here offer a protocol to ascertain the capability of wild yeasts for removing mycotoxins and heavy metals. It has been confirmed once again that microbial detoxification is strain- and toxin-dependent. The best performing species in regard to AFB_1_ detoxification also showed biocontrol activity against *A. parasiticus* (H1—*A. pullulans* and EW1—*K. pastoris*). This finding, along with tolerance to Zn and its biodetoxification, could mean that some of the studied yeast strains will be of great interest for the treatment of contaminated environments, e.g. AK11 (*C. pastoris*), H1, EB39 (*Rh. mucilaginosa*), and EB83 (*S. cerevisiae*) among others. Finally, results obtained from the cell viability test supported the idea that other pathways, such as bioaccumulation or biosorption, may be used by yeast for eliminating contaminant compounds. Consequently, future studies will be carried out in this regard.
